# Impact of non-steroidal anti-inflammatory drugs on malignant transformation in oral lichen planus: insights from a real-world cohort study

**DOI:** 10.3389/fphar.2026.1752108

**Published:** 2026-02-10

**Authors:** Christian Seebauer, Ralf Ludwig, Peter Sieg, Henning Olbrich, Philip Curman

**Affiliations:** 1 Department of Oral and Maxillofacial Surgery / Plastic surgery, University of Lübeck, Lübeck, Germany; 2 Center for Clinical Plasma Research and Therapy, University of Lübeck, Lübeck, Germany; 3 Institute of Experimental Dermatology, University of Lübeck, Lübeck, Germany; 4 Department of Dermatology, Allergy, and Venerology, University of Lübeck, Lübeck, Germany; 5 Comprehensive Center for Inflammation Medicine, University of Lübeck, Lübeck, Germany; 6 Dermato-Venereology Clinic, Karolinska University Hospital, Stockholm, Sweden; 7 Department of Medical Epidemiology and Biostatistics, Karolinska Institutet, Stockholm, Sweden; 8 Dermatology and Venereology Division, Department of Medicine (Solna), Karolinska Institutet, Stockholm, Sweden

**Keywords:** corticosteroids, immunosuppressants, malignant transformation, oral lichen planus, oral squamous cell carcinoma

## Abstract

**Introduction:**

Oral lichen planus (OLP) is a chronic inflammatory condition with malignant potential for oral squamous cell carcinoma (OSCC). Differential risks of pharmacological treatments, particularly long-term use, remain unclear. We aimed to quantify OSCC risk across treatment modalities and assess potential benefit of combining immunosuppressive and anti-inflammatory agents to inform safer strategies.

**Methods:**

We conducted a large, retrospective cohort study with propensity score matching to balance demographic and clinical covariates. Patients with OLP treated with either systemic or topical glucocorticoids, calcineurin inhibitors, non-steroidal anti-inflammatory drugs (NSAIDs), or combinations were followed. OSCC incidence rates were compared between treatment groups, controlling for confounders and stratifying by route and duration of therapy.

**Results:**

Compared with glucocorticoid regimens, calcineurin inhibitors were associated with higher OSCC risk (glucocorticoids: HR 1.85, 95% CI 1.48–2.32; calcineurin inhibitors: HR 3.17, 1.48–6.76). The lowest risk was seen with topical glucocorticoids. Concomitant NSAIDs, particularly ketorolac and diclofenac, with topical glucocorticoids or calcineurin inhibitors, reduced OSCC risk (topical glucocorticoids: HR 0.73, 0.58–0.91; plus ketorolac: HR 0.63, 0.38–1.04; topical calcineurin inhibitors alone: HR 1.53, 1.03–2.28; plus ketorolac: HR 0.28, 0.15–0.54). Owing to retrospective design and reliance on ICD-10 coding, residual confounding and misclassification cannot be excluded.

**Conclusion:**

Calcineurin inhibitors carry high risk for malignant transformation in OLP, while topical glucocorticoids, especially NSAID/glucocorticoid combinations, offer safer profiles. These findings call for re-evaluation of treatment guidelines and prospective trials assessing novel or combination therapeutics that optimize long-term safety and symptom control in OLP.

## 1 Introduction

Oral lichen planus (OLP) is a chronic, T-cell-mediated inflammatory disorder of the oral mucosa with an unclear etiology, presenting with a diverse array of clinical manifestations (Roopashree et al., 2010). The pathogenesis of OLP is characterized by a complex immune response to unidentified antigens, though its precise cause remains unknown. The World Health Organization (WHO) classifies OLP as a potentially premalignant lesion due to its risk of malignant transformation (Gale, 2005). Reported frequencies of malignant transformation vary, with most studies indicating rates between 0.4% and 5% over periods ranging from 0.5 to over 20 years (Al-Hashimi et al., 2007; Alrashdan et al., 2016; Boch et al., 2021). Higher rates are typically observed in patients with atrophic-erosive forms of OLP. Despite this, detailed information regarding tumor localization and entities remains scarce, highlighting the need for extensive cohort studies (Georgakopoulou et al., 2012). Management of OLP remains symptomatic, as no curative treatment currently exists. Therapeutic strategies focus on symptom alleviation and quality-of-life improvement. Given its immune-mediated pathogenesis, treatments typically involve anti-inflammatory, immunomodulatory, and immunosuppressive agents. Glucocorticoids, particularly topically applied glucocorticoids, represent the primary therapeutic option, effectively reducing symptoms by suppressing key inflammatory mediators and modulating cell-mediated immunity (Gupta and Jawanda, 2015; Ismail et al., 2007; Louisy et al., 2024; Nosratzehi, 2018; Kadmiel and Cidlowski, 2013). However, prolonged glucocorticoid use is associated with significant adverse effects across multiple organ systems, including osteoporosis, pneumonia, cardiovascular diseases, renal impairment, and type 2 diabetes (Price et al., 2018; Kelly et al., 2008; Fuhlbrigge and Sharma, 2021).

Alternative second- and third-line therapies include topical calcineurin inhibitors, systemic glucocorticoids, systemic retinoids, immunomodulators, herbal rinses, and laser therapy (Louisy et al., 2024; da Silva et al., 2021; Ślebioda and Dorocka-Bobkowska, 2020). Immunosuppressive medications require cautious use due to the potential malignant transformation risk in OLP patients. Currently, conclusive evidence regarding specific treatments affecting malignant transformation risk is lacking (Georgakopoulou et al., 2012; Ismail et al., 2007; da Silva et al., 2021; Bindakhil et al., 2022; Ezzatt and Helmy, 2019; Lodi et al., 2020; Sieg et al., 1995).

The role of non-steroidal anti-inflammatory drugs (NSAIDs) in OLP management remains controversial. Previous literature suggests that NSAIDs may precipitate or exacerbate erosive lesions and lichenoid reactions, indicating a potential detrimental effect, while clinical improvement has been observed in some cases following NSAID withdrawal. However, NSAIDs also exhibit anti-inflammatory properties, which could theoretically benefit OLP management by modulating inflammation and possibly reducing malignant transformation risk (Andabak-Rogulj et al., 2023; Hamburger and Potts, 1983; Potts et al., 1987; Singh et al., 2017).

To date, no studies have explicitly investigated the role of NSAIDs on oral squamous cell carcinoma (OSCC) development in OLP, despite OLP being recognized as a potentially precancerous lesion. Given the known anti-inflammatory and immunomodulatory properties of commonly used medications, including glucocorticoids and calcineurin inhibitors, NSAIDs could thoretically play a beneficial role in managing OLP and reducing malignant transformation risk.

This retrospective cohort study examines the long-term effects and potential influence of NSAIDs on the development of OSCC arising from the precancerous mucosal disorder OLP. Our analysis aims to provide comprehensive insights into the real-world impact of NSAIDs, potentially challenging traditional therapeutic recommendations.

## 2 Materials and methods

### 2.1 Study design and database

**FIGURE 1:**
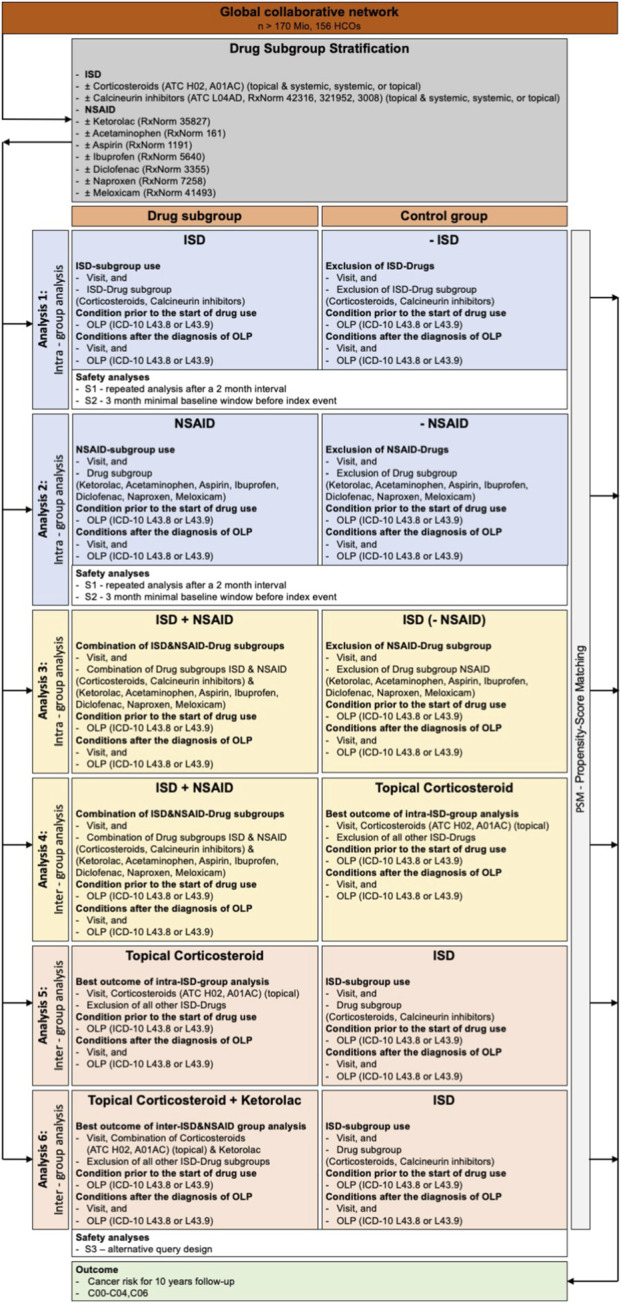
Graphical representation of the study design structure.

We conducted a global, propensity score matched (PSM), retrospective cohort study utilizing real-world data, inspired by previously published investigations (Ludwig et al., 2025; Olbrich et al., 2024; Seebauer et al., 2025). The study used electronic health records (EHR) from the Global Collaborative Network of the federated TriNetX platform. This network was selected for its extensive global reach, encompassing over 170 million patient records from 156 healthcare organizations (HCOs) at the time of analysis (Palchuk et al., 2023). Patients with and without OLP were identified using ICD-10 codes. Symptom-level clinical parameters such as burning sensation, mucosal tenderness, pain intensity scales, or other patient-reported outcome measures were not available in a structured or harmonized format within the TriNetX platform and therefore could not be included in the analysis. Study outcomes were predefined prior to data acquisition and evaluated following PSM. The overall study design is depicted in Figure 1. A comprehensive overview of all diagnostic and treatment codes used is provided in Supplementary Table S1.

### 2.2 Study population and outcome analysis

Data collection and analysis were conducted in July 2025. Six analytical stages were designed to systematically evaluate the OSCC risk associated with the two most frequently prescribed immunosuppressive drugs (ISDs) for OLP, namely, glucocorticoids and calcineurin inhibitors, as well as the potential risk modification associated with the combined use of these ISDs and NSAIDs.

To this end, patient subgroups were created based on medication exposure, utilizing standardized drug identifiers. Medication exposure reflects documented prescriptions or medication orders rather than confirmed intake. Detailed information on dose, cumulative exposure, treatment duration, and adherence was not available. Patients with OLP were identified using ICD-10-CM codes L43.8 and L43.9, under the assumption that cutaneous-only variants of lichen planus, which are rare, would more likely be coded under L43.0 or L43.1. In medication-related analyses, ISD administration routes were classified as topical, systemic, or combined topical and systemic, to account for differential exposure patterns. For NSAID analysis, route categorization was omitted to avoid excessively small subgroup sizes.

Inclusion criteria for all medication subgroups required a confirmed diagnosis of OLP both before and after the index date, documentation of at least one follow-up visit thereafter, and initiation of the respective medication at least 2 weeks after diagnosis to exclude short-term or single-course treatments.

In the first two analytical stages, intra-group comparisons were conducted to assess individual OSCC risks associated with either ISDs (Analysis 1) or NSAIDs (Analysis 2). For Analysis 1, the ISD group included patients treated with either glucocorticoids (ATC: H02, A01AC) or calcineurin inhibitors (ATC: L04AD, RxNorm: 42316, 321952, 3008). For Analysis 2, the NSAID group comprised patients exposed to non-steroidal anti-inflammatory drugs such as ketorolac (RxNorm: 35827), paracetamol (RxNorm: 161), ibuprofen (RxNorm: 5640), acetylsalicylic acid (ASA) (RxNorm: 1191), diclofenac (RxNorm: 3355), naproxen (RxNorm: 7258), and meloxicam (RxNorm: 41493). In both analyses, each medication-exposed subgroup was compared to an OLP comparator subgroup without exposure to the respective drug. Intra-group comparisons were performed to identify the medication associated with the most favorable outcome, defined as the lowest observed OSCC risk. For each agent, patients exposed to the drug were compared to those not exposed to that specific agent.

In the third analytical stage (Analysis 3), intra-group comparisons were conducted to assess OSCC risk across combinations of NSAIDs and ISDs administered via different routes. Each ISD/NSAID combination was compared to the corresponding ISD monotherapy subgroup, excluding patients exposed to the respective NSAID, to identify the most favorable combination in terms of OSCC risk reduction.

In the fourth analytical stage (Analysis 4, ISD/NSAID-specific combinations and their administration routes were compared to the best-performing ISD monotherapy group (from Analysis 1), again excluding patients exposed to other ISDs or NSAIDs.

The fifth analytical stage (Analysis 5) involved inter-group comparisons, where each ISD-specific subgroup was compared directly to the subgroup receiving the best-performing ISD identified in Analysis 1. Patients exposed to any other ISDs were excluded from these comparisons.

In the sixth analytical stage (Analysis 6), inter-group comparisons were performed between glucocorticoid and calcineurin inhibitor subgroups, stratified by administration route, and the subgroup receiving the best-performing ISD/NSAID combination from Analyses 3 and 5. Patients with any additional ISD exposure were excluded.

A comprehensive overview of the study design is provided in Figure 1.

The primary outcome of OSCC was defined by a documentation of any the following ICD-10-CM codes: C00, C01, C02, C03, C04, or C06. Palatal lesions (C05) were excluded because they are uncommon, whereas the lip (C00), tongue (C01–C02), gingiva (C03), floor of the mouth (C04), and buccal mucosa (C06) represent the most frequent sites of OLP (Mollaoglu, 2000).

To assess the robustness of our findings, three sensitivity analyses were performed: (S1) repetition of the entire analysis after imposing a 2-month latency period (lag window); (S2) restriction to patients with a minimum baseline observation of at least 3 months prior to the index date to mitigate detection bias and ensure sufficient data availability for comorbidity profiling and PSM in Analyses 1 and 2; and (S3) verification of the reliability of the top results from Analysis 6 through an alternative query design in which OLP was defined as a “first-instance” diagnosis.

### 2.3 Covariates

To account for potential confounders, PSM was performed, incorporating clinically relevant variables from the domains of demographics, inflammatory diseases, cardiovascular conditions, internal medicine, mental and behavioral disorders, neurological diseases, oral health and dental treatments, medication use, as well as socioeconomic and psychosocial factors. Propensity scores were estimated using logistic regression, and 1:1 matching was conducted using the greedy nearest-neighbor algorithm with a caliper width of 0.1 standard deviations. Baseline characteristics and covariate distributions were compared between groups before and after PSM. Detailed definitions of all covariates are provided in Table 1.

**TABLE 1:** Baseline characteristics of patients with Oral Lichen Planus (OLP) and non-OLP comparators before and after propensity score matching (PSM).

Class	Definition	Code	Before matching	​	​	​	After matching	​	​	​
​	​	​	OLP [n = 40,988]	Healthy comparators [n = 155,792]	p	Standardized difference	OLP [n = 30,202]	Healthy comparators [n = 30,202]	p	Standardized difference
Demographics	Age at index [years]	​	58.7 ± 15.8 (38,785)	31.4 ± 24.1 (154,440)	<0.0001	1.3385	55.7 ± 16 (30,202)	57.2 ± 18.2 (30,202)	<0.0001	0.0861
	Sex (female)	​	71.388% (27,688)	53.297% (82,312)	<0.0001	0.3801	68.121% (20,574)	65.78% (19,867)	<0.0001	0.0498
	White	​	63.12% (24,481)	41.35% (63,861)	<0.0001	0.4466	61.837% (18,676)	62.734% (18,947)	0.0229	0.0185
	Black or African American	​	11.615% (4,505)	27.155% (41,938)	<0.0001	0.4009	13.582% (4,102)	14.069% (4,249)	0.0831	0.0141
	Hispanic or Latino	​	4.607% (1,787)	14.43% (22,286)	<0.0001	0.3395	5.609% (1,694)	5.864% (1,771)	0.1779	0.0110
	Not Hispanic or Latino	​	68.903% (26,724)	76.093% (117,518)	<0.0001	0.1615	74.442% (22,483)	77.68% (23,461)	<0.0001	0.0759
	Asian	​	5.409% (2,098)	13.221% (20,419)	<0.0001	0.2712	6.781% (2,048)	7.288% (2,201)	0.0149	0.0198
Inflammatory diseases	Thyroiditis	E06	1.9% (688)	0.6% (880)	<0.0001	0.122	1.8% (494)	0.9% (252)	<0.001	0.077
	Keratitis	H16	1.8% (637)	1.0% (1,542)	<0.0001	0.068	1.5% (415)	1.6% (428)	0.652	0.004
	Iridocyclitis	H20	0.6% (209)	0.4% (699)	0.0007	0.019	0.6% (160)	0.6% (158)	0.910	0.001
	Asthma	J45	10.0% (3,607)	11.8% (18,524)	<0.0001	0.057	10.3% (2,785)	9.1% (2,466)	<0.001	0.040
	Atopic dermatitis	L20	2.7% (956)	6.0% (9,374)	<0.0001	0.163	2.9% (777)	2.2% (597)	<0.001	0.042
	Esophagitis	K20	2.2% (790)	1.4% (2,257)	<0.0001	0.057	2.1% (568)	1.5% (396)	<0.001	0.048
	Gastritis and duodenitis	K29	5.5% (1,987)	4.6% (7,161)	<0.0001	0.044	5.3% (1,442)	5.7% (1,537)	0.073	0.015
	Noninfective enteritis and colitis	K50-K52	7.8% (2,798)	8.2% (12,834)	0.0129	0.015	7.7% (2,080)	6.0% (1,637)	<0.001	0.065
	Other inflammatory liver diseases	K75	1.5% (537)	1.2% (1,825)	<0.0001	0.029	1.5% (395)	1.2% (334)	0.023	0.020
Cardiovascular diseases	Hypertensive diseases	I10-I1A	33.1% (11,934)	17.6% (27,656)	<0.0001	0.363	30.9% (8,359)	30.9% (8,372)	0.904	0.001
	Essential (primary) hypertension	I10	32.7% (11,795)	17.2% (27,086)	<0.0001	0.364	30.5% (8,264)	30.6% (8,280)	0.881	0.001
	Angina pectoris	I20	2.2% 8,785)	2.0% (3,224)	0.1229	0.009	2.1% (568)	3.1% (839)	<0.0001	0.063
	Chronic ischemic heart disease	I25	7.3% (2,622)	3.7% (85,885)	<0.0001	0.155	6.7% (1,812)	6.1% (1,646)	0.004	0.025
	Heart failure	I50	3.0% (1,064)	2.1% (3,324)	<0.0001	0.053	3.1% (829)	3.0% (812)	0.670	0.004
	Cerebrovascular diseases	I60-I69	5.3% (1,913)	3.7% (5,872)	<0.0001	0.076	4.7% (1,277)	5.6% (1,520)	<0.001	0.041
	Cerebral infarction	I63	1.7% (604)	1.3% (2,076)	<0.0001	0.029	1.5% (416)	2.2% (584)	<0.001	0.046
	Atherosclerosis	I70	2.5% (897)	1.1% (1,698)	<0.0001	0.107	2.2% (587)	2.1% (576)	0.744	0.003
	Other peripheral vascular diseases	I73	3.2% (1,153)	1.3% (2,082)	<0.0001	0.126	2.9% (790)	2.5% (676)	0.003	0.026
	Other venous embolism and thrombosis	I82	1.9% (697)	1.1% (1,714)	<0.0001	0.069	1.9% (524)	1.5% (416)	<0.001	0.031
Internal diseases	Body mass index	39,156–5	29.1 ± 6.8 (18,276)	26.9 ± 7.9 (91,227)	<0.0001	0.303	29.2 ± 7.0 (13,874)	28.7 ± 7.1 (18,505)	<0.001	0.077
	Diabetes mellitus	E08-E13	13.3% (4,809)	8.3% (12,979)	<0.0001	0.165	13.0% (3,521)	12.9% (3,495)	0.739	0.003
	Overweight, obesity and other hyperalimentation	E65-E68	15.0% (5,401)	15.0% (23,522)	0.8801	0.001	15.2% (4,129)	15.6% (4,213)	0.317	0.009
	Metabolic syndrome	E88.810	0.2% (80)	0.2% (268)	0.0372	0.012	0.2% (60)	0.2% (55)	0.641	0.004
	Overweight and obesity	E66	14.8% (5,322)	14.8% (23,266)	0.9039	0.001	15.1% (4,079)	15.3% (4,146)	0.422	0.007
	Chronic lower respiratory diseases	J40-J4A	15.9% (5,740)	15.7% (24,741)	0.3546	0.005	15.9% (4,309)	15.2% (4,105)	0.016	0.021
	Neoplasms	C00-D49	36.0% (12,974)	19.8% (31,156)	<0.0001	0.367	34.5% (9,344)	32.3% (8,744)	<0.001	0.047
	Chronic viral hepatitis	B18	1.7% (596)	1.3% (1,997)	<0.0001	0.032	1.7% (469)	1.7% (458)	0.716	0.003
	Chronic viral hepatitis B with delta-agent	B18.0	0.0% (10)	0.0% (28)	0.2244	0.007	0.0% (10)	0.0% (10)	1	<0.001
	Chronic viral hepatitis B without delta-agent	B18.1	0.3% (95)	0.4% (663)	<0.0001	0.027	0.3% (85)	0.3% (72)	0.299	0.009
	Human immunodeficiency virus [HIV] disease	B20	0.5% (195)	1.5% (2,364)	<0.0001	0.096	0.7% (189)	0.7% (202)	0.509	0.006
	Diseases of the digestive system	K00-K95	48.7% (17,545)	55.6% (87,465)	<0.0001	0.139	48.1% (13,022)	47.5% (12,871)	0.194	0.011
	Fibrosis and cirrhosis of liver	K74	1.6% (573)	1.0% (1,641)	<0.0001	0.048	1.6% (431)	1.1% (296)	<0.001	0.043
	Chronic kidney disease (CKD)	N18	4.3% (1,558)	3.3% (5,121)	<0.0001	0.056	4.2% (1,131)	3.9% (1,043)	0.054	0.017
Mental and behavioral disorders and diseases of the brain	Mental disorders due to known physiological conditions	F01-F09	1.7% (601)	2.3% (3,661)	<0.0001	0.047	2.0% (532)	2.0% (550)	0.580	0.005
	Alcohol related disorders	F10	2.2% (775)	2.4% (3,753)	0.0076	0.016	2.4% (660)	2.6% (692)	0.378	0.008
	Nicotine dependence	F17	6.6% (82,364)	5.6% (8,878)	<0.0001	0.038	7.1% (1,935)	7.7% (2,085)	0.014	0.021
	Other psychoactive substance dependence with other psychoactive	F19.28	0.0% (10)	0.0% (41)	0.8594	0.001	0.0% (10)	0.0% (10)	1	<0.001
	Schizophrenia, schizotypal, delusional, and other non-mood psychotic disorders	F20-F29	0.9% (332)	1.7% (2,713)	<0.0001	0.070	1.0% (277)	1.7% (466)	<0.001	0.060
	Manic episode	F30	0.1% (38)	0.1% (222)	0.0953	0.010	0.1% (33)	0.1% (34)	0.903	0.001
	Bipolar disorder	F31	1.4% (502)	1.7% (2,660)	<0.0001	0.024	1.6% (438)	1.7% (467)	0.331	0.008
	Depressive episode	F32	13.3% (4,786)	10.8% (16,986)	<0.0001	0.076	13.4% (3,642)	14.1% (3,830)	0.019	0.020
	Major depressive disorder, recurrent	F33	3.6% (1,314)	3.6% (5,720)	0.9321	<0.001	4.0% (1,076)	4.2% (1,141)	0.159	0.012
	Persistent mood [affective] disorders	F34	2.5% (892)	1.7% (2,634)	<0.0001	0.056	2.4% (654)	2.5% (666)	0.738	0.003
	Phobic anxiety disorders	F40	0.7% (266)	0.9% (1,451)	0.0008	0.020	0.8% (212)	0.8% (221)	0.664	0.004
	Other anxiety disorders	F41	15.1% (5,455)	12.3% (19,332)	<0.0001	0.083	14.8% (4,005)	14.2% (3,854)	0.065	0.016
	Reaction to severe stress, and adjustment disorders	F43	5.4% (1,955)	7.0% (11,056)	<0.0001	0.066	5.6% (1,519)	5.8% (1,576)	0.291	0.009
	Dissociative and conversion disorders	F44	0.3% (111)	0.3% (452)	0.5123	0.004	0.3% (90)	0.4% (98)	0.559	0.005
	Somatoform disorders	F45	1.1% (404)	1.3% (2,084)	0.0019	0.019	1.2% (328)	1.2% (323)	0.844	0.002
	Anxiety, dissociative, stress-related, somatoform and other nonpsychotic mental disorders	F40-F48	18.4% (6,644)	17.7% (27,809)	<0.0001	0.020	18.2% (4,936)	18.4% (4,978)	0.641	0.004
	Behavioral syndromes associated with physiological disturbances and physical factors	F50-F59	3.5% (1,269)	3.7% (5,844)	0.0770	0.010	3.7% (999)	3.5% (937)	0.151	0.012
	Disorders of adult personality and behavior	F60-F69	0.6% (219)	2.3% (3,688)	<0.0001	0.144	0.7% (196)	0.7% (197)	0.960	<0.001
	Intellectual disabilities	F70-F79	0.2% (63)	3.0% (4,693)	<0.0001	0.227	0.2% (63)	0.2% (59)	0.717	0.003
	Multiple sclerosis	G35	0.5% (173)	0.2% (365)	<0.0001	0.042	0.5% (128)	0.4% (109)	0.216	0.011
	Sleep disorders	G47	15.1% (5,435)	14.6% (22,888)	<0.0001	0.015	15.0% (4,050)	17.1% (4,629)	<0.001	0.058
Oral health and dental treatments	Gingivitis and periodontal diseases	K05	1.9% (702)	11.4% (17,912)	<0.0001	0.385	2.5% (670)	2.4% (647)	0.521	0.006
	Dental caries	K02	1.9% (700)	23.0% (36,158)	<0.0001	0.672	2.6% (700)	2.5% (682)	0.624	0.004
	Amalgam restorations	D2140-D2161	0.1% (42)	6.6% (10,307)	<0.0001	0.364	0.1% (38)	8.8% (2,382)	<0.001	0.428
	Resin-based composite restorations	D2330-D2394	0.3% (112)	27.3% (42,983)	<0.0001	0.851	0.4% (110)	28.3% (7,675)	<0.001	0.868
	Oral examination	122,856,003	0.1% (52)	8.9% (13,940)	<0.0001	0.430	0.2% (52)	10.1% (2,737)	<0.001	0.460
	Dental prophylaxis	D1110-D1120	0.5% (183)	91.1% (143,357)	<0.0001	4.375	0.6% (173)	89.9% (24,350)	<0.001	4.049
	Endodontics	D3110-D3999	0.1% (45)	6.7% (10,573)	<0.0001	0.369	0.2% (45)	0.3% (69)	0.024	0.019
	Periodontics	D4210-D4999	0.2% (58)	4.7% (7,422)	<0.0001	0.299	0.2% (58)	0.3% (68)	0.372	0.008
	Prosthodontics (removable)	D5110-D5899	0.1% (40)	3.3% (5,224)	<0.0001	0.249	0.1% (40)	0.2% (57)	0.084	0.015
	Implant services	D6010-D6199	0.1% (19)	1.1% (1,683)	<0.0001	0.136	0.1% (19)	0.1% (22)	0.639	0.004
	Gold foil restorations	D2410-D2430	0%	0%	​	​	0%	0%	​	​
Medications	Corticosteroids for systemic use	H02	50.1% (18,050)	42.9% (67,550)	<0.0001	0.143	50.4% (13,658)	40.8% (11,049)	<0.001	0.194
	Corticosteroids for local oral treatment	A01AC	39.9% (14,375)	37.8% (59,495)	<0.0001	0.042	40.1% (10,870)	33.8% (9,163)	<0.001	0.131
	Calcineurin inhibitors	L04AD	6.3% (2,255)	1.6% (2,479)	<0.0001	0.243	6.1% (1,662)	1.9% (511)	<0.001	0.218
	Tacrolimus	42,316	4.5% (1,616)	1.1% (1,665)	<0.0001	0.210	4.6% (1,243)	1.0% (269)	<0.001	0.220
	Cyclosporine	3008	2.1% (769)	0.6% (980)	<0.0001	0.130	1.9% (510)	1.0% (267)	<0.001	0.076
	Pimecrolimus	321,952	1.1% (407)	0.8% (1,182)	<0.0001	0.039	1.1% (305)	0.7% (200)	<0.001	0.040
	Acetaminophen	161	34.9% (13,558)	45.9% (70,967)	<0.0001	0.2254	35.9% (10,839)	35.7% (10,769)	0.5524	0.0048
	Ibuprofen	5640	16.3% (6,322)	33.4% (51,624)	<0.0001	0.4043	18.2% (5,483)	18.4% (5,560)	0.4176	0.0066
	Aspirin	1191	16.9% (6,557)	8.6% (13,322)	<0.0001	0.2501	15.4% (4,644)	15.4% (4,663)	0.8304	0.0017
	Ketorolac	35,827	12.5% (4,838)	14.1% (21,819)	<0.0001	0.0487	12.7% (3,839)	12.9% (3,891)	0.5265	0.0052
	Naproxen	7258	9.2% (3,553)	9.9% (15,340)	<0.0001	0.0263	10.1% (3,060)	10.5% (3,160)	0.1806	0.0109
	Diclofenac	3355	9.1% (3,530)	8.8% (13,596)	0.0648	0.0104	9.5% (2,866)	9.4% (2,853)	0.8566	0.0015
	Meloxicam	41,493	7.4% (2,851)	3.5% (5,356)	<0.0001	0.1723	6.7% (2,026)	6.9% (2,082)	0.3654	0.0074
Socioeconomic and psychosocial circumstances	Problems related to employment and unemployment	Z56	0.2% (70)	0.2% (372)	0.1296	0.009	0.2% (61)	0.2% (67)	0.595	0.005
	Problems related to housing and economic circumstances	Z59	0.4% (144)	1.4% (2,153)	<0.0001	0.104	0.5% (138)	0.5% (143)	0.765	0.003
	Problems related to life management difficulty	Z73	0.2% (70)	1.0% (1,649)	<0.0001	0.109	0.2% (56)	0.7% (186)	<0.001	0.072
	Family history of primary malignant neoplasm	Z80	7.4% (2,677)	3.5% (5,546)	<0.0001	0.172	6.7% (1,822)	7.1% (6.7%)	0.068	0.016
	Personal history of nicotine dependence	Z87.891	6.9% (2,471)	3.0% (4,728)	<0.0001	0.179	5.9% (1,599)	6.2% (1,674)	0.176	0.012

p-values ≤0.05 were considered statistically significant. SMD, standardized mean difference; SD, standard deviation. An SMD >0.1 was interpreted as indicative of relevant imbalance and a potentially meaningful difference between groups and is highlighted in bold. § Covariates included in the PSM.

### 2.4 Statistical analysis

OSCC outcomes were analyzed up to 10 years following the index event, defined as the registration of OLP or treatment initiation. The first month after the index event was excluded to better avoid including individuals with preexisting conditions. EHRs with outcomes occurring before the index event were also excluded from all analyses. Relative risks and risk differences were calculated, and survival analyses were conducted using Kaplan-Meier curves. Log-rank tests were used to compare Kaplan-Meier curves, and hazard ratios (HRs) with 95% confidence intervals (CIs) were derived from univariate Cox regression analyses. p-values below 0.05 were considered significant. Patients were censored at their last recorded follow-up. Covariate balance was assessed using standardized mean differences (SMD), with values below 0.1 considered indicative of adequate balance between matched cohorts. Owing to technical constraints of the TriNetX platform, only pairwise 1:1 PSM using a greedy nearest-neighbor algorithm with a predefined caliper (0.1 pooled standard deviations of the logit of the propensity score) was feasible.

### 2.5 Ethics statement

This study represents a secondary analysis of pre-existing data and did not involve any direct intervention or interaction with human subjects. Consequently, Institutional Review Board (IRB) approval was not required, as all data were obtained in aggregated and de-identified form through the TriNetX database. The de-identification process strictly adheres to the requirements of the U.S. HIPAA Privacy Rule (§164.514[a]) and applies the Expert Determination methodology mandated by the U.S. Department of Health and Human Services (HHS). As such, the data are not considered Protected Health Information (PHI) and therefore fall outside the scope of the Privacy Rule, eliminating the need for additional ethics committee approval or oversight (Ludwig et al., 2025; Seebauer et al., 2025; Kuo et al., 2024).

The study was conducted in accordance with established ethical principles, including the Declaration of Helsinki, the U.S. Federal Policy for the Protection of Human Subjects (45 CFR 46), and the European Medicines Agency Guidelines for Good Clinical Practice, and followed the STROBE reporting guidelines (Cuschieri, 2019). In addition, the study protocol received approval from the Swedish Ethical Review Authority (diary number 2025-03805-02).

## 3 Results

### 3.1 Baseline characteristics

We identified 40,988 EHRs with a documented OLP and 155,792 non-OLP comparators. Prior to PSM, the OLP cohort was substantially older (58.7 ± 15.8 years) compared to the comparator cohort (31.4 ± 24.1 years), corresponding to a SMD of 1.34. A female predominance was observed in both groups (71.4% vs. 53.3%; SMD = 0.38), and the majority of patients in both cohorts were White (63.1% vs. 41.4%; SMD = 0.45). Following PSM, the cohorts showed balanced distributions across key demographic variables indicating adequate covariate balance (SMD <0.1). A detailed overview of the cohorts’ characteristics, including all covariates included in the regression model as well as baseline characteristics before and after matching, is presented in Table 1.

### 3.2 Analysis 1: efficacy of ISD monotherapy

**FIGURE 2:**
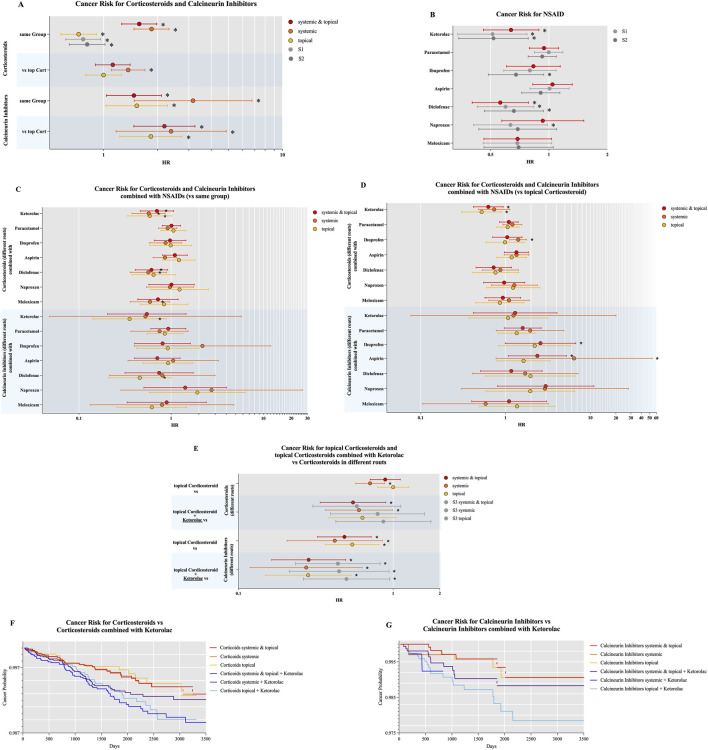
Presents the results of the oral squamous cell carcinoma risk analysis for: **(A)** immunosuppressive drugs (ISDs) alone, compared to no ISD treatment (white background) and to topical glucocorticoids (blue background); **(B)** non-steroidal anti-inflammatory drugs (NSAIDs) alone; **(C)** combination therapy of ISDs and NSAIDs compared to ISDs alone (glucocorticoids: white background; calcineurin inhibitors: blue background); **(D)** combination therapy of ISDs and NSAIDs compared to topical glucocorticoids (glucocorticoids: white background; calcineurin inhibitors: blue background); **(E–G)** topical glucocorticoids compared to ISDs (without ketorolac: white background; with ketorolac: blue background). The figure includes forest plots and Kaplan–Meier survival curves to illustrate hazard ratios and time-to-event distributions. Bars marked with an asterisk indicate statistically significant results (p ≤ 0.05).

Topical glucocorticoid monotherapy was associated with a significantly reduced OSCC risk in patients with OLP (HR 0.73, 95% CI 0.58–0.91, p = 0.0058), compared to both systemic glucocorticoid treatment (HR 1.85, 1.48–2.32) and treatment with calcineurin inhibitors (topical: HR 1.53, 1.03–2.28; systemic: HR 3.17, 1.48–6.76). Among all immunosuppressive drugs evaluated in Analysis 1, topical glucocorticoids demonstrated the most favorable outcome, with the lowest associated OSCC risk. A comprehensive summary of these results is presented in Table 2 and Figure 2A (white background).

**TABLE 2:** Presents the oral squamous cell carcinoma risks associated with the immunospuuressive drugs administered glucocorticoids.

Drug	Route	Patients in OLP cohort, n (comparators)	Patients with outcome, n (comparators)	Risk (%)	HR	95% CI		p
Glucocorticoids	Systemic and topical	34,546 (34,745)	200 (120)	0.579 (0.345)	1.583	1.263	1.986	<0.0001
	Systemic	32,672 (33,072)	212 (117)	0.649 (0.354)	1.854	1.479	2.324	<0.0001
	Topical	27,122 (27,068)	130 (169)	0.479 (0.624)	0.726	0.577	0.912	0.0058
	S1	28,292 (28,232)	138 (169)	0.488 (0.599)	0.769	0.614	0.963	0.0215
	S2	28,668 (28,598)	135 (158)	0.471 (0.552)	0.81	0.644	1.02	0.0725
Calcineurin inhibitors	Systemic and topical	7,595 (7,567)	72 (54)	0.948 (0.714)	1.478	1.038	2.104	0.0293
	Systemic	1,942 (1,936)	26 (≤10)	1.339 (0.517)	3.167	1.483	6.764	0.0017
	Topical	6,966 (6,948)	59 (42)	0.847 (0.604)	1.534	1.032	2.28	0.0329

### 3.3 Analysis 2: efficacy of NSAID monotherapy

Monotherapy with either diclofenac (HR 0.56, 0.40–0.79) or ketorolac (HR 0.64, 0.46–0.89) was associated with a significantly reduced OSCC risk compared to other tested NSAIDs, including paracetamol, ibuprofen, acetylsalicylic acid (ASA), naproxen, and meloxicam. Among all NSAIDs evaluated in Analysis 2, diclofenac and ketorolac demonstrated the most favorable outcomes, with the lowest associated OSCC risks. A comprehensive summary of these findings is presented in Table 3 and Figure 2B.

**TABLE 3:** Presents the oral squamous cell carcinoma risks associated with the non-steroidal anti-inflammatory drugs (NSAIDs) ketorolac, paracetamol, ibuprofen, aspirin, diclofenac, naproxen, and meloxicam, as well as the results of the sensitivity analysis for these NSAIDs.

Drug	Patients in OLP cohort, n (comparators)	Patients with outcome, n (comparators)	Risk (%)	HR	95% CI		p
Ketorolac	16,562 (16,611)	55 (99)	0.332 (0.596)	0.638	0.459	0.888	0.0072
S1	11,821 (11,862)	33 (75)	0.279 (0.632)	0.512	0.339	0.771	0.0011
S2	11,458 (11,485)	33 (73)	0.288 (0.636)	0.518	0.343	0.783	0.0015
Paracetamol	38,996 (39,448)	239 (262)	0.613 (0.664)	0.944	0.792	1.124	0.5167
S1	41,391 (41,872)	264 (277)	0.638 (0.662)	0.998	0.843	1.181	0.9775
S2	40,288 (40,781)	259 (288)	0.643 (0.706)	0.924	0.782	1.093	0.3591
Ibuprofen	15,375 (15,619)	69 (84)	0.449 (0.538)	0.832	0.605	1.144	0.2575
S1	16,311 (16,534)	71 (90)	0.435 (0.544)	0.798	0.585	1.089	0.1539
S2	14,598 (14,817)	60 (88)	0.411 (0.594)	0.677	0.487	0.94	0.0189
ASA	19,686 (19,895)	142 (138)	0.721 (0.694)	1.044	0.826	1.32	0.7161
S1	19,608 (19,808)	142 (143)	0.724 (0.722)	1.008	0.799	1.271	0.9481
S2	19,241 (19,449)	142 (156)	0.738 (0.802)	0.907	0.723	1.139	0.4006
Diclofenac	13,183 (13,238)	53 (98)	0.402 (0.74)	0.562	0.402	0.785	0.0006
S1	13,220 (13,296)	54 (93)	0.408 (0.699)	0.598	0.427	0.836	0.0024
S2	12,356 (12,419)	52 (78)	0.421 (0.628)	0.66	0.464	0.937	0.0192
Naproxen	7,772 (7,759)	33 (32)	0.425 (0.412)	0.93	0.572	1.513	0.7708
S1	8,196 (8,207)	34 (49)	0.415 (0.597)	0.634	0.409	0.982	0.0394
S2	7,425 (7,425)	32 (41)	0.431 (0.552)	0.691	0.435	1.097	0.1150
Meloxicam	9,762 (9,759)	39 (61)	0.4 (0.625)	0.69	0.462	1.032	0.0690
S1	9,303 (9,305)	38 (60)	0.408 (0.645)	0.688	0.458	1.034	0.0704
S2	8,928 (8,919)	38 (57)	0.426 (0.639)	0.698	0.463	1.053	0.0846

### 3.4 Analysis 3: efficacy of ISD + NSAID combination therapy: intra-group analysis comparing NSAID exposure to non-exposure

The concomitant use of NSAIDs with ISDs was associated with a significant reduction in OSCC risk compared to ISD monotherapy for the treatment of OLP. In particular, the NSAIDs ketorolac (topical: HR 0.59, 0.34–1.02; systemic: HR 0.57, 0.38–0.86), diclofenac (systemic: HR 0.57, 0.36–0.89), and meloxicam (systemic: HR 0.59, 0.36–0.96) demonstrated a significantly reduced OSCC risk when combined with glucocorticoids.

For patients treated with calcineurin inhibitors, the combination with ketorolac (topical: HR 0.35, 0.14–0.89) and diclofenac (topical: HR 0.46, 0.21–1.00) was also associated with a significantly reduced OSCC risk. A comprehensive summary of these findings is presented in Table 4 and Figure 2C.

**TABLE 4:** Presents the oral squamous cell carcinoma risks associated with combination therapy of ISDs and NSAIDs, compared to ISD monotherapy excluding NSAID use.

Drug	Drug	Route	Patients in OLP cohort, n (comparators)	Patients with outcome, n (comparators)	Risk (%)	HR	95% CI		p
Ketorolac	Glucocorticoids	Systemic and topical	12,877 (12,908)	36 (58)	0.28 (0.449)	0.701	0.462	1.063	0.0931
		Systemic	11,873 (11,884)	34 (66)	0.286 (0.555)	0.57	0.377	0.863	0.0071
		Topical	7,386 (7,406)	19 (37)	0.257 (0.5)	0.585	0.337	1.019	0.0552
	Calcineurin inhibitors	Systemic and topical	1,698 (1,713)	≤10 (12)	0.589 (0.701)	0.545	0.204	1.451	0.2171
		Systemic	394 (396)	≤10 (≤10)	2.538 (2.525)	0.525	0.048	5.786	0.5919
		Topical	1,679 (1,700)	≤10 (19)	0.596 (1.118)	0.354	0.141	0.886	0.0203
Paracetamol	Glucocorticoids	Systemic and topical	26,624 (26,813)	145 (149)	0.545 (0.556)	1.008	0.802	1.267	0.9460
		Systemic	23,583 (23,747)	138 (155)	0.585 (0.653)	0.917	0.729	1.154	0.4594
		Topical	15,464 (15,533)	80 (78)	0.517 (0.502)	1.061	0.777	1.449	0.7099
	Calcineurin inhibitors	Systemic and topical	3,613 (3,645)	38 (42)	1.052 (1.152)	0.932	0.601	1.446	0.7544
		Systemic	940 (950)	13 (18)	1.383 (1.895)	0.749	0.367	1.528	0.4252
		Topical	3,458 (3,484)	35 (42)	1.012 (1.206)	0.855	0.546	1.339	0.4939
Ibuprofen	Glucocorticoids	Systemic and topical	10,604 (10,743)	48 (50)	0.453 (0.465)	0.973	0.655	1.446	0.8922
		Systemic	9,204 (9,331)	45 (53)	0.489 (0.568)	0.87	0.585	1.295	0.4935
		Topical	6,883 (6,928)	28 (28)	0.407 (0.404)	0.989	0.585	1.669	0.9658
	Calcineurin inhibitors	Systemic and topical	1,647 (1,670)	14 (18)	0.85 (1.078)	0.807	0.402	1.623	0.5475
		Systemic	387 (397)	≤10 (≤10)	2.584 (2.519)	2.185	0.4	11.931	0.3547
		Topical	1,589 (1,605)	12 (13)	0.755 (0.81)	0.919	0.419	2.014	0.8327
ASA	Glucocorticoids	Systemic and topical	12,998 (13,117)	84 (78)	0.646 (0.595)	1.099	0.808	1.496	0.5474
		Systemic	11,515 (11,612)	79 (95)	0.686 (0.818)	0.857	0.636	1.155	0.3098
		Topical	8,065 (8,109)	51 (43)	0.632 (0.53)	1.217	0.811	1.827	0.3411
	Calcineurin inhibitors	Systemic and topical	1,801 (1,826)	20 (29)	1.11 (1.588)	0.708	0.4	1.251	0.2323
		Systemic	488 (499)	≤10 (≤10)	2.049 (2.004)	1.053	0.34	3.266	0.9287
		Topical	1,613 (1,631)	19 (21)	1.178 (1.288)	0.928	0.499	1.726	0.8127
Diclofenac	Glucocorticoids	Systemic and topical	10,139 (10,137)	37 (62)	0.365 (0.612)	0.611	0.407	0.918	0.0167
		Systemic	7,857 (7,857)	29 (52)	0.369 (0.662)	0.565	0.359	0.89	0.0125
		Topical	6,703 (6,697)	21 (34)	0.313 (0.508)	0.644	0.374	1.109	0.1099
	Calcineurin inhibitors	Systemic and topical	1,385 (1,395)	≤10 (13)	0.722 (0.932)	0.74	0.316	1.733	0.4866
		Systemic	293 (290)	≤10 (≤10)	3.413 (3.448)	0.802	0.215	2.989	0.7418
		Topical	1,616 (1,626)	≤10 (20)	0.619 (1.23)	0.456	0.208	1.002	0.0450
Naproxen	Glucocorticoids	Systemic and topical	5,568 (5,558)	25 (23)	0.449 (0.414)	1.01	0.573	1.779	0.9730
		Systemic	4,862 (4,854)	23 (22)	0.473 (0.453)	0.971	0.541	1.742	0.9215
		Topical	3,841 (3,836)	17 (13)	0.443 (0.339)	1.233	0.599	2.54	0.5684
	Calcineurin inhibitors	Systemic and topical	849 (849)	≤10 (≤10)	1.178 (1.178)	1.422	0.506	3.995	0.5023
		Systemic	157 (158)	≤10 (≤10)	6.369 (6.329)	2.7061	0.286	26.462	0.3611
		Topical	834 (836)	≤10 (≤10)	1.199 (1.196)	1.923	0.579	6.387	0.2773
Meloxicam	Glucocorticoids	Systemic and topical	6,251 (6,242)	25 (38)	0.4 (0.609)	0.719	0.434	1.191	0.1983
		Systemic	5,611 (5,592)	25 (44)	0.446 (0.787)	0.589	0.361	0.963	0.0327
		Topical	4,541 (4,536)	19 (25)	0.418 (0.551)	0.836	0.46	1.519	0.5570
	Calcineurin inhibitors	Systemic and topical	1,059 (1,068)	≤10 (≤10)	0.944 (0.936)	0.895	0.333	2.407	0.8261
		Systemic	219 (221)	≤10 (≤10)	4.566 (4.525)	0.795	0.133	4.764	0.8005
		Topical	1,048 (1,053)	≤10 (14)	0.954 (1.33)	0.618	0.259	1.473	0.2725

Notably, combining ketorolac with glucocorticoids reduced the OSCC risk from HR 0.73 (topical monotherapy) to HR 0.58 (p < 0.05), and from HR 1.85 (systemic glucocorticoids alone) to HR 0.57 (p < 0.05). Similarly, the combination of ketorolac with calcineurin inhibitors reduced the OSCC risk from HR 1.53 to HR 0.35 (topical, p < 0.05) and from HR 3.17 to HR 0.53 (systemic, p < 0.05).

Combining diclofenac with glucocorticoids lowered the OSCC risk from HR 1.85 to HR 0.57 (systemic, p < 0.05), while its combination with topical calcineurin inhibitors reduced the risk from HR 1.53 to HR 0.46 (p < 0.05).

These findings are best understood by comparing Table 2 (ISD monotherapy) with Table 4 (ISD + NSAID combination therapy).

### 3.5 Analysis 4: efficacy of ISD + NSAID combination therapy: inter-group analysis comparing NSAID exposure to topical glucocorticoid s (best monotherapy Outcome)

Compared to topical glucocorticoid monotherapy, the most favorable treatment identified in Analysis 1 with the lowest associated OSCC risk, the combination of glucocorticoids with ketorolac was the only regimen to demonstrate a further and statistically significant reduction in OSCC risk (topical combination: HR 0.52, 0.30–0.90). These findings are best appreciated by comparing Table 2 (ISD monotherapy) with Table 5 (ISD + NSAID combination therapy vs. topical glucocorticoid).

**TABLE 5:** Presents the oral squamous cell carcinoma risks associated with combination therapy of ISDs and NSAIDs, compared to topical glucocorticoids identified as the best-performing treatment in Analysis 1.

Drug	Drug	Route	Patients in OLP cohort, n (comparators)	Patients with outcome, n (comparators)	Risk (%)	HR	95% CI		p
Ketorolac	Glucocorticoids	Systemic and topical	12,859 (12,901)	36 (68)	0.28 (0.527)	0.627	0.418	0.939	0.0223
		Systemic	11,860 (11,913)	34 (56)	0.287 (0.47)	0.736	0.48	1.129	0.1585
		Topical	7,386 (7,406)	19 (42)	0.257 (0.567)	0.523	0.304	0.9	0.0172
	Calcineurin inhibitors	Systemic and topical	1,699 (1,710)	≤10 (≤10)	0.589 (0.585)	1.301	0.418	4.047	0.6487
		Systemic	392 (395)	≤10 (≤10)	2.551 (2.532)	1.245	0.077	20.008	0.8770
		Topical	1,677 (1,693)	≤10 (≤10)	0.596 (0.591)	1.074	0.371	3.11	0.8950
Paracetamol	Glucocorticoids	Systemic and topical	23,214 (23,540)	123 (119)	0.53 (0.506)	1.097	0.853	1.412	0.4712
		Systemic	21,473 (21,821)	124 (113)	0.577 (0.518)	1.208	0.936	1.558	0.1465
		Topical	15,464 (15,533)	80 (78)	0.517 (0.502)	1.061	0.777	1.449	0.7099
	Calcineurin inhibitors	Systemic and topical	3,617 (3,649)	38 (26)	1.051 (0.713)	1.598	0.97	2.632	0.0632
		Systemic	1,022 (1,036)	13 (≤10)	1272 (0.965)	1.957	0.781	4.904	0.1447
		Topical	3,464 (3,491)	35 (28)	1.01 (0.802)	1.359	0.827	2.234	0.2249
Ibuprofen	Glucocorticoids	Systemic and topical	10,598 (10,766)	48 (47)	0.453 (0.437)	1.049	0.702	1.569	0.8152
		Systemic	26,345 (26,521)	169 (128)	0.641 (0.483)	1.413	1.123	1.777	0.0031
		Topical	6,883 (6,928)	28 (28)	0.407 (0.404)	0.989	0.585	1.669	0.9658
	Calcineurin inhibitors	Systemic and topical	1,649 (1,672)	14 (≤10)	0.849 (0.598)	2.588	0.994	6.738	0.0432
		Systemic	389 (400)	≤10 (0)	2.571 (0)	​	​	​	​
		Topical	1,589 (1,612)	12 (≤10)	0.755 (0.62)	2.214	0.831	5.901	0.1028
ASS	Glucocorticoids	Systemic and topical	12,759 (12,924)	82 (64)	0.643 (0.495)	1.349	0.973	1.871	0.0716
		Systemic	11,313 (11,490)	77 (62)	0.681 (0.54)	1.352	0.968	1.889	0.0761
		Topical	8,064 (8,102)	51 (44)	0.632 (0.543)	1.184	0.791	1.773	0.4101
	Calcineurin inhibitors	Systemic and topical	1,805 (1,825)	20 (≤10)	1.108 (0.548)	2.379	1.083	5.226	0.0259
		Systemic	527 (538)	≤10 (≤10)	1.898 (1.859)	6.439	0.775	53.485	0.0472
		Topical	1,685 81,702)	19 (12)	1.128 (0.705)	1.636	0.794	3.371	0.1774
Diclofenac	Glucocorticoids	Systemic and topical	8,805 (8,862)	28 (41)	0.318 (0.463)	0.726	0.449	1.174	0.1899
		Systemic	7,852 (7,914)	29 (36)	0.369 (0.455)	0.872	0.534	1.422	0.5816
		Topical	5,879 (5,877)	17 (23)	0.289 (0.391)	0.765	0.409	1.433	0.4016
	Calcineurin inhibitors	Systemic and topical	1,624 (1,630)	11 (11)	0.677 (0.675)	1.171	0.507	2.707	0.7116
		Systemic	366 (366)	≤10 (≤10)	2.732 (2.732)	1.712	0.409	7.164	0.4565
		Topical	1,349 (1,348)	≤10 (≤10)	0.741 (0.742)	1.973	0.576	6.751	0.2701
Naproxen	Glucocorticoids	Systemic and topical	5,568 (5,558)	25 (24)	0.449 (0.432)	0.966	0.552	1.692	0.9035
		Systemic	4,861 (4,861)	23 (17)	0.473 (0.35)	1.273	0.68	2.382	0.4502
		Topical	3,841 (3,836)	17 (13)	0.443 (0.339)	1.233	0.599	2.54	0.5684
	Calcineurin inhibitors	Systemic and topical	850 (851)	≤10 (≤10)	1.176 (1.175)	2.978	0.806	11	0.0856
		Systemic	159 (158)	≤10 (≤10)	6.289 (6.329)	2.921	0.304	28.083	0.3303
		Topical	835 (835)	≤10 (≤10)	1.198 (1.198)	1.963	0.591	6.52	0.2617
Meloxicam	Glucocorticoids	Systemic and topical	7,291 (7,274)	30 (36)	0.411 (0.495)	**0.933**	**0.574**	**1.515**	0.7782
		Systemic	5,610 (5,586)	25 (26)	0.446 (0.465)	1.101	0.635	1.907	0.7322
		Topical	3,948 (3,932)	16 (20)	0.405 (0.509)	0.86	0.446	1.661	0.6538
	Calcineurin inhibitors	Systemic and topical	1,109 (1,106)	≤10 (≤10)	0.902 (0.904)	1.103	0.399	3.05	0.8494
		Systemic	255 (258)	≤10 (≤10)	3.922 (3.876)	0.583	0.106	3.188	0.5284
		Topical	1,069 (1,063)	≤10 (≤10)	0.935 (0.941)	1.371	0.496	3.792	0.5412

### 3.6 Analysis 5: efficacy of topical glucocorticoid monotherapy: inter-group analysis comparing topical glucocorticoid (best monotherapy outcome) to other ISD

Topical glucocorticoid therapy was associated with a significantly lower OSCC risk compared to both systemic glucocorticoids (HR 0.71, 0.57–0.88) and calcineurin inhibitors administered either topically (HR 0.54, 0.36–0.81) or systemically (HR 0.42, 0.21–0.85). A comprehensive summary of these findings is presented in Table 6 and Figure 2E (white background).

**TABLE 6:** Presents the oral squamous cell carcinoma risk of topically administered glucocorticoids in comparison to other immunosuppressive drugs (ISDs) across various routes of administration.

Drug	Route	Patients in OLP cohort, n (comparators)	Patients with outcome, n (comparators)	Risk (%)	HR	95% CI		p
Glucocorticoids	Systemic and topical	29,770 (29,768)	146 (163)	0.49 (0.548)	0.888	0.71	1.11	0.2951
	Systemic	29,688 (29,502)	146 (193)	0.492 (0.654)	0.709	0.572	0.879	0.0016
	Topical	29,772 (29,772)	146 (146)	0.49 (0.49)	1	​	​	​
Calcineurin inhibitors	Systemic and topical	7,787 (7,808)	37 (70)	0.475 (0.897)	0.484	0.325	0.721	0.0003
	Systemic	1,958 (1,953)	11 (25)	0.562 (1.28)	0.419	0.206	0.852	0.0132
	Topical	7,448 (7,485)	38 (65)	0.51 (0.868)	0.544	0.364	0.811	0.0024

### 3.7 Analysis 6: efficacy of topical glucocorticoid + ketorolac combination therapy: inter-group analysis comparing topical glucocorticoid (best monotherapy outcome) combined with ketorolac to other ISD

The combination of ketorolac with topical glucocorticoids, representing the best-performing treatment in terms of OSCC risk reduction, further decreased the OSCC risk when compared to glucocorticoid or calcineurin inhibitor therapy without ketorolac. Specifically, OSCC risk was reduced compared to topical glucocorticoids alone (from HR 1.00 to HR 0.63, 0.38–1.04) and systemic glucocorticoids (from HR 0.71 to HR 0.60, 0.36–0.99).

A similar risk reduction was observed when comparing the ketorolac-glucocorticoid combination to calcineurin inhibitors: from HR 0.54 to HR 0.28 for topical application (0.15–0.54), and from HR 0.42 to HR 0.27 for systemic application (0.12–0.63).

These findings are best interpreted by comparing Table 6 (topical glucocorticoids vs. glucocorticoids and calcineurin inhibitors) with Table 7 (ketorolac plus topical glucocorticoids vs. glucocorticoids and calcineurin inhibitors without ketorolac). A comprehensive overview of these results is provided in Figures 2E–G.

**TABLE 7:** Presents the oral squamous cell carcinoma risk associated with combined topical glucocorticoid and ketorolac therapy in comparison with other immunosuppressive drugs (ISDs) administered via different routes, as well as the results of sensitivity analysis S3.

Drug	Route	Patients in OLP cohort, n (comparators)	Patients with outcome, n (comparators)	Risk (%)	HR	95% CI		p
Glucocorticoids	Systemic and topical	8,109 (8,131)	24 (49)	0.296 (0.603)	**0.55**	0.337	0.897	0.0150
	S3	3,434 (3,424)	14 (25)	0.408 (0.73)	**0.582**	0.302	1.12	0.1009
	Systemic	8,109 (8,123)	24 (43)	0.296 (0.529)	**0.601**	0.364	0.99	0.0432
	S3	3,434 (3,430)	14 (18)	0.408 (0.525)	**0.793**	0.394	1.594	0.5133
	Topical	8,076 (8,095)	24 (43)	0.297 (0.531)	**0.633**	0.384	1.043	0.0703
	S3	3,434 (3,432)	14 (17)	0.408 (0.495)	**0.864**	0.426	1.754	0.6860
Calcineurin inhibitors	Systemic and topical	5,666 (5,702)	16 (59)	0.282 (1.035)	**0.284**	0.164	0.494	<0.0001
	S3	3,405 (3,401)	14 (32)	0.411 (0.941)	**0.439**	0.234	0.823	0.0082
	Systemic	2,013 (2,023)	≤10 (27)	0.497 (1.335)	**0.274**	0.119	0.63	0.0011
	S3	2,394 (2,377)	≤10 (23)	0.418 (0.968)	**0.447**	0.213	0.94	0.0293
	Topical	4,744 (4,785)	12 (44)	0.253 (0.92)	**0.282**	0.149	0.535	<0.0001
	S3	3,371 (3,366)	14 (28)	0.415 (0.832)	**0.499**	0.263	0.948	0.0303

Bolded values represent the hazard ratios.

## 4 Discussion

Our study shows that the risk of malignant transformation in OLP is strongly influenced by the pharmacological agent used. The large, propensity score matched cohort and long-term follow-up allowed us to demonstrate that calcineurin inhibitors are associated with a substantially higher risk of OSCC compared with glucocorticoids, with the lowest risk observed for topically administered glucocorticoids. While treatment choice and route of administration are frequently determined by individual efficacy and symptom relief, the main drivers for seeking medical care and initiating therapy, our findings highlight a critical limitation: alternative immunosuppressive therapies may not mitigate, and in the case of calcineurin inhibitors may even increase, carcinogenic risk. These results underscore the urgent need for novel therapeutic approaches, including the development and rigorous evaluation of alternative agents or combination regimes that combine anti-inflammatory and immunosuppressive mechanisms to ensure both long-term efficacy and safety.

Chronic inflammation is increasingly acknowledged as a key driver of carcinogenesis, with OLP representing a prototypical condition prone to malignant transformation arising from persistent inflammatory processes (Alrashdan et al., 2016; Deng et al., 2009; Giagkou et al., 2016). Owing to its immune-mediated pathogenesis, current therapeutic strategies for OLP primarily rely on anti-inflammatory, immunomodulatory, and immunosuppressive interventions (Kurago, 2016; Lavanya et al., 2011). Among immunosuppressive agents, glucocorticoids and calcineurin inhibitors represent the most commonly used pharmacologic therapies, due to their well-established efficacy in controlling local inflammation and immune activation (Al-Hashimi et al., 2007; Bindakhil et al., 2022; Becker et al., 2006).

### 4.1 Potential synergistic effect of NSAIDs and topical immunosuppressive drugs in the treatment of chronic inflammatory mucosal diseases

NSAIDs may represent a valuable adjunct in the treatment of chronic inflammatory mucosal diseases when combined with topical ISDs such as glucocorticoids and calcineurin inhibitors. NSAIDs exert their anti-inflammatory effects primarily through inhibition of cyclooxygenase (COX) enzymes, thereby reducing prostaglandin synthesis and modulating key inflammatory pathways involved in mucosal damage and pain.

When used in combination, NSAIDs and ISDs may act synergistically by targeting distinct and complementary mechanisms within the inflammatory cascade. While glucocorticoids and calcineurin inhibitors broadly suppress immune activation, by downregulating pro-inflammatory cytokine expression, T-cell activity, and leukocyte infiltration, NSAIDs predominantly attenuate prostaglandin-mediated vascular responses, edema, and nociception. This dual mechanism may enhance anti-inflammatory efficacy, improve local symptom control, and allow for dose reduction of ISDs, thereby minimizing the risk of adverse effects associated with long-term immunosuppressive therapy.

NSAIDs exhibit heterogeneous anti-inflammatory properties based on their selectivity for COX isoenzymes (Andabak-Rogulj et al., 2023). While agents such as ibuprofen, diclofenac, ketorolac, and aspirin act on both COX-1 and COX-2, meloxicam demonstrates a preferential selectivity for COX-2. In contrast, paracetamol, although often grouped among non-opioid analgesics, lacks significant anti-inflammatory activity due to its minimal peripheral COX inhibition (Hamburger and Potts, 1983).

These pharmacological differences are reflected in our findings. In our analyses, paracetamol showed no measurable additive effect on OSCC risk reduction when used in combination with ISDs. In contrast, COX-1/2 inhibitors such as diclofenac and ketorolac were associated with a notable decrease in OSCC risk when combined with either glucocorticoids or calcineurin inhibitors.

Among all NSAIDs evaluated, ketorolac emerged as the agent with the most pronounced risk-reducing effect. It significantly lowered the OSCC risk associated with both classes of ISDs, and this effect was particularly remarkable in combination with topically administered glucocorticoids, which on their own already exhibited the lowest OSCC risk among the tested immunosuppressants. This suggests a potentially synergistic interaction that warrants further mechanistic and clinical investigation.

Ketorolac is available in multiple formulations, including topical preparations, which opens avenues for local combination therapies. For example, a dual-application regimen using a glucocorticoid-containing mouth rinse alongside a ketorolac mouth rinse could be considered. Alternatively, the development of a combined ketorolac–glucocorticoid formulation may represent a promising strategy for maximizing therapeutic efficacy while minimizing systemic exposure and associated risks.

To date, no comparable study has systematically addressed the potential synergistic effect between immunosuppressive and anti-inflammatory agents in the context of cancer risk modulation. While a few studies have investigated the use of NSAIDs in inflammatory mucosal conditions, the existing literature remains limited and does not adequately explore their role in combination with immunosuppressive therapies (Andabak-Rogulj et al., 2023; Hamburger and Potts, 1983; Potts et al., 1987; Singh et al., 2017).

### 4.2 Strengths and limitations

This study represents the largest exploratory real-world investigation to date assessing pharmacological risk modification of OSCC in patients with OLP and the first to systematically evaluate combinations of immunosuppressive drugs and NSAIDs. The large sample size, global coverage, long follow-up, and use of PSM to balance a broad range of demographic and clinical covariates constitute key strengths. Several important limitations must be acknowledged.

First, the retrospective design inherently limits causal inference, and residual confounding cannot be fully excluded despite extensive PSM. In particular, confounding by indication remains a relevant concern, as information on OLP subtype and disease severity was not available. Treatment selection may therefore reflect baseline malignant potential rather than a direct therapeutic effect.

Second, case identification relied on ICD-10-CM codes (L43.8 and L43.9) due to the absence of a dedicated diagnostic code for OLP. While this represents a necessary and widely used approach in large-scale EHR-based research, some degree of misclassification and overlap with cutaneous lichen planus cannot be excluded. Restricting outcomes to intraoral malignancies partially mitigates this limitation but does not eliminate it.

Third, medication exposure was defined based on documented prescriptions or medication orders. Detailed information on dose, cumulative exposure, treatment adherence, and confirmed intake was not available. NSAID exposure is likely underascertained due to over-the-counter availability, which may bias effect estimates toward the null.

Fourth, symptom-level clinical parameters and patient-reported outcome measures, such as pain severity, burning sensation, and oral discomfort, were not available in a structured form. As symptom burden frequently drives treatment selection in OLP, the absence of these data may influence interpretation of observed associations.

Additionally, several HR estimates, particularly for combination therapies, were associated with wide confidence intervals, occasionally including unity, reflecting limited precision and underscoring the exploratory nature of the findings.

Finally, the cohort was predominantly composed of older individuals, which may limit generalizability to younger OLP populations, even though key risk factors were addressed through PSM.

## 5 Conclusion

In this large, propensity score-matched real-world cohort study, we observed that the risk of malignant transformation in OLP varies substantially across pharmacological treatment strategies. Calcineurin inhibitors were associated with a higher incidence of OSCC, whereas topically administered glucocorticoids demonstrated the most favorable safety profile. Selected NSAID, particularly ketorolac and diclofenac, were associated with signals of lower OSCC risk when used in combination with immunosuppressive therapies, with the combination of topical glucocorticoids and ketorolac showing the most pronounced risk reduction.

Given the retrospective design, reliance on diagnostic coding, and limited clinical granularity inherent to large EHR-based datasets, these findings should be interpreted as exploratory and hypothesis-generating rather than causal. In particular, residual confounding and confounding by indication cannot be excluded, as treatment selection may reflect underlying disease severity and malignant potential.

Nevertheless, the observed associations highlight potentially clinically relevant signals that may inform the design of future prospective, pathology-verified studies. Such studies should incorporate detailed phenotyping of OLP, standardized assessment of disease severity and patient-reported outcome measures, and precise characterization of pharmacological exposure to clarify whether specific treatment strategies can meaningfully reduce the long-term risk of malignant transformation.

## Data Availability

The datasets presented in this study can be found in online repositories. The names of the repository/repositories and accession number(s) can be found in the article/Supplementary Material.
