# Supplementary material for: Impact of non-steroidal anti-inflammatory drugs on malignant transformation in oral lichen planus: insights from a real-world cohort study
Source: Front Pharmacol. 2026 Feb 10;17:1752108. doi: 10.3389/fphar.2026.1752108 (PMC12929388; doi:10.3389/fphar.2026.1752108)
Supplement: Supplementary file 1 [file Table1.docx]

Supplementary Material

| **Inclusion criteria** |  |  |
| --- | --- | --- |
| Description | Identifiers | Identifier description |
| Oral lichen planus | ICD-10-CM: L43.8, L43.9 | Other lichen planus; lichen planus, unspecified |
| Visit | Visit | Visit |
| Glucocorticoids | ATC: H02, A01AC (restricted to topical, injectable, and oral products) | Corticosteroids for systemic use; corticosteroids for local oral treatment |
| Calcineurin inhibitors | ATC: L04AD, RxNorm: 42316, 321952, 3008 (restricted to topical, injectable, and oral products) | Calcineurin inhibitors, tacrolimus, pimecrolimus, Cyclosporine |
| Ketorolac | RxNorm: 35827 | Ketorolac |
| Acetaminophen | RxNorm: 161 | Paracetamol |
| Aspirin | RxNorm: 1191 | Acetylsalicylic acid |
| Ibuprofen | RxNorm: 5640 | Ibuprofen |
| Diclofenac | RxNorm: 3355 | Diclofenac |
| Naproxen | RxNorm: 7258 | Naproxen |
| Meloxicam | RxNorm: 41493 | Meloxicam |
| **Outcomes** |  |  |
| Description | Identifiers | Identifier description |
| Oral squamous cell carcinoma | ICD-10-CM: C00, C01, C02, C03, C04, C06 | Malignant neoplasm of lip, malignant neoplasm of base of tongue, malignant neoplasm of other and unspecified parts of tongue, malignant neoplasm of gum, malignant neoplasm of floor of mouth, malignant neoplasm of other and unspecified parts of mouth |

**Supplementary Table 1** List of registration codes and identifiers used in the study. Unless otherwise specified, codes are based on ICD-10-CM, RxNorm, and ATC classifications.
